# Unraveling Presenilin 2 Functions in a Knockout Zebrafish Line to Shed Light into Alzheimer’s Disease Pathogenesis

**DOI:** 10.3390/cells12030376

**Published:** 2023-01-19

**Authors:** Lucia Barazzuol, Domenico Cieri, Nicola Facchinello, Tito Calì, Philip Washbourne, Francesco Argenton, Paola Pizzo

**Affiliations:** 1 Department of Biomedical Sciences, University of Padova, 35131 Padua, Italy; 2 Neuroscience Institute, National Research Council (CNR), 35131 Padua, Italy; 3 Centro Studi per la Neurodegenerazione (CESNE), University of Padova, 35131 Padua, Italy; 4 Institute of Neuroscience, University of Oregon, Eugene, OR 97403, USA; 5 Department of Biology, University of Padova, 35131 Padua, Italy

**Keywords:** presenilin 2, Alzheimer’s disease, zebrafish, ER-mitochondria contacts, mitochondrial axonal transport, autophagy

## Abstract

Mutations in presenilin 2 (PS2) have been causally linked to the development of inherited Alzheimer’s disease (AD). Besides its role as part of the γ-secretase complex, mammalian PS2 is also involved, as an individual protein, in a growing number of cell processes, which result altered in AD. To gain more insight into PS2 (dys)functions, we have generated a *presenilin2* (*psen2*) knockout zebrafish line. We found that the absence of the protein does not markedly influence Notch signaling at early developmental stages, suggesting a Psen2 dispensable role in the γ-secretase-mediated Notch processing. Instead, loss of Psen2 induces an exaggerated locomotor response to stimulation in fish larvae, a reduced number of ER-mitochondria contacts in zebrafish neurons, and an increased basal autophagy. Moreover, the protein is involved in mitochondrial axonal transport, since its acute downregulation reduces in vivo organelle flux in zebrafish sensory neurons. Importantly, the expression of a human AD-linked mutant of the protein increases this vital process. Overall, our results confirm zebrafish as a good model organism for investigating PS2 functions in vivo, representing an alternative tool for the characterization of new AD-linked defective cell pathways and the testing of possible correcting drugs.

## 1. Introduction

Alzheimer’s disease (AD) is the most common cause of age-related dementia worldwide. Approximately 2% of AD is inherited in an autosomal dominant fashion and presents an early age of onset and a more rapid rate of progression [1,2]. Gain-of-function mutations in *APP* (encoding amyloid precursor protein (APP)), *PSEN1* (encoding presenilin 1 (PS1)), and *PSEN2* (encoding presenilin 2 (PS2)) account for almost all cases of dominantly inherited AD.

PSs are multi−transmembrane proteins that form the catalytic subunit of the γ-secretase complex, an intramembrane aspartyl-protease complex that cleaves an array of type-1 transmembrane substrates, of which APP is one of the most widely studied given its connection to AD. Interestingly, to date, there are nearly 300 potentially pathogenic mutations identified in PS1, but less than 60 in PS2 [3]. It is still unclear whether all the pathogenic mutations act similarly. However, it appears that, although the two PS proteins are highly homologous (they share 62% identity at the amino acid residue sequence), they can differentially contribute to several biological processes. For example, the fact that PS2-containing γ-secretase complexes have been described to be mainly targeted to the late endosome/lysosome compartment, differently from those containing PS1, may underlie the subtly different function/specificity of the two paralogs [4].

Since their identification, PSs have also been found to be involved in biological functions independently of their γ-secretase activity, including cellular calcium (Ca^2+^) homeostasis, mitochondrial metabolism, autophagy, and protein transport [5]. Notably, both PSs have been found enriched at mitochondria-associated membranes (MAMs) [6], suggesting their involvement in ER-mitochondria communication. Interestingly, PS2, but not PS1, was found to alter ER-mitochondria juxtaposition [7,8] by interacting with mitofusin 2 (MFN2), a well-known player in ER-mitochondria tethering [9]. The mechanisms underlying this unique PS2 function, and its potential effects in neuronal physiopathology, are still partially unclear. What is known, however, is that the protein is involved in neuronal viability; it influences several cellular processes that have been found altered in AD. For example, AD-PS2 mutants reduce the Ca^2+^ content of intracellular stores [10,11,12,13,14], as well as store-operated Ca^2+^ entry [15]. Moreover, the expression of mutant PS2 is associated with impaired autophagy [16], defective mitochondrial activity [17], and cell bioenergetics [18].

Because of several advantageous characteristics, zebrafish has become one of the most prominent vertebrate model organisms used to study the genetics underlying development, normal body functions, and diseases. As zebrafish shows high genome conservation with humans, it is not surprising that the components of the γ-secretase signaling pathway have been identified in this model organism. Both the human *PSEN1* and *PSEN2* genes have orthologs in zebrafish, *psen1* and *psen2*, respectively [19,20]. The couples of ortholog proteins, specifically, share more than 70% amino acid sequence identity [19,20]. The other components of the γ-secretase complex, Aph-1, Pen-2, and nicastrin, have also been identified in zebrafish [21,22].

Different strategies have been applied to specifically investigate *psen* roles in zebrafish physiology, starting with the acute knockdown (KD) of the proteins. Focusing on *psen2*, reducing the expression of the protein (Psen2) by morpholino injection gives rise to a fish phenotype resembling the KD of *psen1*. Specifically, both phenotypes strongly point to Notch signaling dysregulation, i.e., somite defects, increased neurogenesis, altered expression pattern of Notch target genes, reduced pigmentation, short tail, smaller head, and defective brain development [23,24]. However, an increase in the number of dorsal longitudinal ascending interneurons was only observed in the *psen2* acute KD fish, suggesting the existence of nonredundant roles for both Psen proteins in zebrafish [23]. In addition, different stable *psen2* mutant lines have been generated. The full knockout (KO) of *psen2* (*psen2^uaa7/uaa7^*) impacts pigmentation, as Psen2 presence seems to be specifically required for normal adult melanotic skin pigmentation [25]. In a *psen2* knockin (KI) line (*psen2^uaa8/uaa8^*), in which the coding frame of the *psen2* gene was conserved [25], the pigmentation defect is milder, suggesting a specific effect of the wild-type (WT) protein on zebrafish physiology.

Overall, the goal of the majority of the published studies on these stable *psen2* mutant zebrafish lines was to identify the cellular processes affected by familial AD-linked mutations in young adult heterozygous mutant brains (specifically searching for an early familial AD brain transcriptomic signature). In this respect, the heterozygous KI mutation (*psen2^uaa8/+^*) uniquely caused subtle, but statistically significant, changes to the expression of genes involved in oxidative phosphorylation, long-term potentiation, and cell cycle. Differently, the heterozygous KO mutation (*psen2^ua78/+^)* uniquely affected genes involved in Notch and MAPK signaling, extracellular matrix receptor interactions, and focal adhesion [26]. Further, both types of mutation affected ribosomal protein gene expression, but in opposite directions [26]. Less has been performed to deeper characterize the homozygous KO line in order to gain more insight into protein physiological roles, which are still partially unclear.

To specifically study these aspects of Psen2, we generated and characterized a *psen2* full KO line; the line results to be a valid tool to study Psen2 physiology, investigating specific protein functions that have been linked to PS2 in mammals. Moreover, the line is also very useful to study, in a null background, the effect of human AD-linked PS2 mutants in vivo on multiple processes, avoiding toxic unspecific outcomes possibly linked to both morpholino and protein-overexpressing approaches.

## 2. Materials and Methods

### 2.1. Animals

All experiments were performed in accordance with the European and Italian Legislations and with permission for animal experimentation from the Local Ethics Committee of the University of Padova (OPBA) (Project Number: 269/2020), released in 2020 and valid for 3 years). Embryos were obtained by natural mating and raised at 28.5 °C in Petri dishes containing fish water (50X: 25 g Instant Ocean (Aquarium Systems, SS15-10), 39.25 g CaSO_4_, and 5 g NaHCO_3_ for 1 L) and kept in a 12:12 light-dark (LD) cycle. Adult fish were maintained and raised in 5 L tanks with freshwater at 28 °C with a 12 h light/12 h dark cycle. Nonmutant embryos or larvae indicated as WT or *psen2^+/+^* correspond to samples deriving from different or from the same egg batches of *psen2^−/−^*, respectively.

### 2.2. Generation of psen2^−/−^ Zebrafish Line

The *psen2^−/−^* zebrafish mutants were generated by CRISPR/Cas9-mediated genome editing, as described by Jao et al. [27]. A single-guide RNA (sgRNA) was designed to specifically target an optimal CRISPR sequence on exon 4 of the *psen2* gene. The *psen2*-targeting sgRNA (with the specific targeting sequence GAATTCGGTGCTCAACACTC–tgg) was generated according to [27], using the primers TAGGATTCGGTGCTCAACACTC and AAACGAGTGTTGAGCACCGAAT, and transcribed in vitro using the MEGAshortscript T7 kit (Thermo Fisher Scientific, Waltham, MA, USA). One-cell stage embryos were injected with 1–2 nL of a solution containing 150 ng/µL nls-zCas9-nls mRNA (Addgene, Watertown, MA, USA; Cat# 47927) and 100 ng/µL sgRNA; Phenol Red was used as an injection marker. F0-injected embryos were raised to adulthood and screened by genotyping the F1 for germline transmission of the mutation. *psen2* heterozygous and homozygous mutants were identified by loading in agarose gel the product of the PCR performed using the following primers: *psen2*–forward 5′–CCCCATTTGAAGAAGACCCG–3′ and *psen2*–reverse 5′–AGAGTAAAGCAGGTCTCACTTT–3′, amplifying a 153 bp long DNA fragment containing the deletion. Once *psen2^−/−^* homozygous mutants were isolated, a longer portion of their genomic DNA was amplified using a different forward primer, specifically 5′–TTTCCCAAATAACAAAACAGCCA–3′, in order to allow the sequencing of the PCR products and to confirm the presence of the 7 bp deletion.

### 2.3. Genomic DNA Extraction from Embryos, Larvae, and Fin Clips

Genomic DNA was extracted from single larvae euthanized with a lethal dose of tricaine (Merck KGaA, Darmstadt, Germany) using the HotSHOT protocol [28]. To extract DNA from fin clips, adult fish were anesthetized with tricaine, and biopsies from the caudal fin were removed with a sharp blade. The specimens were then treated with the same protocol.

### 2.4. RNA Isolation and Quantitative Real-Time Reverse Transcription PCR

For expression analysis, total RNA was extracted from pools of 15 larvae or from the brains of adult fish with TRIzol reagent (Thermo Fisher Scientific, Waltham, MA, USA; Cat# 15,596,026). cDNA was synthesized from the RNA using the SuperScript II First-Strand Synthesis System (Invitrogen, Carlsbad, CA, USA) and Random Primers (Promega, Madison, WI, USA, C1181). PCRs were performed with the 2 × qPCRBIO SyGreen Mix Hi-ROX (PCRBIOSYSTEM) in a StepOne™ Real-Time PCR System (Thermo Fisher Scientific, Waltham, MA, USA) machine. Actin was used as an internal standard. The annealing/extension temperature for PCR ranged from 60 to 62 °C, depending on the primer set. The cycling parameters were 95 °C for 2 min, followed by 40 cycles at 95 °C for 5 s, and 30 s of annealing and extension. Melt curve analysis was performed following instrument instructions.

### 2.5. Birefringence Assay

Muscle birefringence was analyzed by placing anesthetized larvae mounted in 2% methylcellulose in H_2_O on a glass polarizing filter and covering them with a second polarizing filter on a Leica M165 FC microscope. Larvae were photographed with a Nikon DS-Fi2 digital camera in bright field. The top filter was twisted to see the light refracting through striated muscle. Pixel intensity in the trunk region was measured with ImageJ software. Values were normalized for the sample area. Birefringence analysis was performed on sibling larvae and was followed by genotyping.

### 2.6. Whole-Mount in Situ Hybridization (WMISH)

Zebrafish embryos and larvae were fixed overnight at 4 °C in 4% paraformaldehyde in phosphate-buffered saline (Merck KGaA, Darmstadt, Germany) at the developmental stages of interest. WMISH was performed as previously described [29].

### 2.7. Generation of Mutant psen2 Transgenic Zebrafish Lines

The *psen2^−/−^* mutant line was crossed with the following transgenic zebrafish lines: *Et(1.5hsp70l:Gal4–VP16)s1102t*: *Tg(UAS–E1b:Kaede)s1999t* and *Tg(EPV.Tpi–Mmu.Hbb:EGFP)^ia^*12. To restrict Gal4 expression to Rohon–Beard (RB) sensory neurons, the already–described *s1102t:Gal4/UAS:Kaede [Et(1.5hsp70l:Gal4–VP16)s1102t; Tg(UAS–E1b:Kaede)s1999t]* zebrafish transgenic line, which constitutively express cytosolic Kaede in RB neurons, was backcrossed with WT fish. Fish positive only for the Gal4 genotype (*s1102t:Gal4*) were raised to adulthood and used for further experiments. To genotype Gal4-positive fish, the primers forward: GTCTCAGCCTCACTTTGAGC and reverse: TGTTAGAGGCATATCAGTCTCCAC were used.

Fluorescence analysis on *Tg(EPV.Tpi–Mmu.Hbb:EGFP)^ia^*12 transgenic background was performed on sibling larvae and was followed by genotyping.

### 2.8. Behavioral Assays

For behavioral assays, 12 8-dpf WT and *psen2^−/−^* larvae were placed in 24-well plates, one larva per well with 2 mL of fish water, and images were recorded with the DanioVision tracking system (Noldus Information Technology, Wageningen, The Netherlands). After 30 min of acclimation, larvae movement was recorded during the administration of a repetitive tapping stimulus (one tap every second for 60 s, maximal intensity of stimulus). Locomotor activity was analyzed using Ethovision 11 software (Noldus Information Technology, Wageningen, The Netherlands).

### 2.9. Generation of Expression Plasmids

In order to target Kaede to mitochondria, a mitochondrial targeting sequence (MTS, from subunit VIII of human cytochrome C oxidase) was introduced at the N–terminus of Kaede by PCR, amplifying Kaede from pME–KAEDE plasmid with the primers forward: 5′-ATTATACCGGATCCATGGTGAGTCTGATTAAACCAGAAATGA-3′ and reverse: 5′-ATTAGCGGCCGCTTACTTGACGTTGTCCGGCAAT-3′. The PCR product was first subcloned into pME–MCS, in order to obtain a middle entry vector (pME–MT–Kaede) to be used with the Gateway^®^ Cloning Technology (Thermo Fisher Scientific, Waltham, MA, USA). The final expression vector was obtained by recombining the entry vectors p5E–UAS, p3E–poly–A, and pME–MT–Kaede with the destination vector pDestTol2pCG2 (Tol–2 kit). A 1–2 nL volume of 30 ng/µL of pTol2–UAS–MT–Kaede (referred to UAS:MT–Kaede hereafter) was then injected into 1-cell stage embryos to obtain transient and mosaic expression of the transgene.

### 2.10. psen2 Knockdown

We performed *psen2* knockdown using the already-described MoPS2Tln morpholino [30]. Morpholino was dissolved in distilled water at a final concentration of 1 mM and injected into fertilized eggs at a 0.3 mM concentration. For rescue experiments, 1–2 nL of 30 ng/µL mRNA transcribed from the TOPO–PS2–WT construct was injected into 1–2 cell stage embryos.

### 2.11. PS2 Overexpression

To overexpress human PS2 in zebrafish embryos, the fragment encoding for human PS2 (wt or T122R) was amplified from pcDNA3–PS2 with primers forward: CACCATGCTCACATTCATGGCCTCTGAC and reverse: AGATCTAGCCATAGAGCCCACCGCAT, then subcloned into pCR^®^–BluntIITOPO^®^. mRNA was generated by BglII linearized templates using the Ambion mMESSAGE mMACHINE^®^ kit (Thermo Fisher Scientific, Waltham, MA, USA). For transport assay, embryos were injected with 1–2 nL of 30 ng/µL PS2 mRNA and pTol2–UAS–MT–Kaede vector.

### 2.12. Transient Expression of SPLICS Probe

To selectively express the SPLICS probe in zebrafish neurons, the Gateway technology was exploited to generate the pT2–DsRed–UAS–SPLICS vector [31], which allows the simultaneous expression of both a cytosolic dsRED to mark neurons and the SPLICS probe. The resulting plasmid was injected into 1–2 cell stage *s1102t:Gal4* embryos (either WT or *psen2^−/−^* embryos in this transgenic background) at a concentration of 50–60 ng/µL. At 24-hpf, embryos were screened for fluorescence, dechorionated and anesthetized. Fish were then mounted on µ-Slide 8-Well Glass Bottom (Ibidi Gmbh, Munich, Germany; Cat# 80,827) in 300 µL/well low-melting agarose (1.2%, EuroClone, Milan, Italy). Fish water containing tricaine methanesulfonate 0.61 mM (Merck KGaA, Darmstadt, Germany) was added above each well, in order to keep fish anesthetized. Mounted fish were imaged at RT (20–23 °C) using a Leica TSC SP5 inverted confocal microscope, using either an HCX PL APO ×63/numerical aperture 1.40–0.60 or an HCX PL APO 100X/numerical aperture 1.4 oil-immersion objective. To count ER-mitochondria contacts, a complete Z-stack of the cell was acquired every 0.5–0.7 μm.

### 2.13. In Vivo Mitochondrial Axonal Transport Imaging

At 24-hpf, embryos were screened for fluorescence. To prepare fish for imaging, they were manually dechorionated, anesthetized using tricaine methanesulfonate 0.61 mM (Merck KGaA, Darmstadt, Germany), and mounted on µ-Slide 8-Well Glass Bottom (Ibidi Gmbh, Munich, Germany; Cat# 80,827) in low-melting agarose (1.2%, EuroClone, Milan, Italy). Embryos were maintained hydrated and anesthetized during imaging by layering the agarose with tricaine-containing fish water. Mounted fish were imaged at room temperature (20–23 °C) using a Leica TSC SP5 inverted confocal microscope, equipped with Ar/ArKr 488 nm and He/Ne 543 nm laser lines and a 405 nm cw 50 mW line, using an HCX PL APO 63X/numerical aperture 1.40–0.60 oil-immersion objective with digital zoom. Time-lapse Z-stacks with slice thickness up to 0.7 μm were acquired with a frame interval between 2–5 s (i.e., a complete Z-stack was acquired in 2–5 s) with a scanning speed of 400 Hz or less and a pinhole of 0.95–1 μm. Axons were imaged for 2 min. Unless otherwise stated, all mitochondrial movement analyses were performed on non-photoconverted larvae, using the green emission channel.

### 2.14. Kymograph Construction and Analysis

Images were processed using Fiji ImageJ software (version 2.9.0/1.53t; Java 1.8.0_172). Videos from Z-stacks were generated by using the “sum slices” option under the Z-project menu. The [Straighten] plugin was used to select the axon from which to generate the kymograph. Kymographs were then generated using the [Reslice] tool with default options. Mitochondrial speeds were calculated using trigonometric functions by measuring the length and angles of lines in the kymographs with the [Measure] tool in ImageJ. Average speed was calculated by measuring the distance covered by each mitochondrion, including pausing times. Mitochondrial flux was measured as the number of moving mitochondria over the number of total mitochondria in the region imaged. Occasionally, the cell body from which the axon had sprouted was easily identified, allowing anterograde and retrograde movements to be discerned by considering the slope of the line from which average speed is calculated. A mitochondrion was considered as moving if its average speed was ≥0.08 μm/s. To determine mitochondrial density, the number of mitochondria was divided over the length of the kymograph in μm. Mitochondrial morphology was evaluated using the ImageJ macro “Morphometry”, previously described by Cribbs and Strack [32]. This macro measures mitochondrial morphology by using two parameters, aspect ratio (the major axis divided by minor axis) and form factor, which take into account the perimeter and area of the organelles, capturing complex mitochondrial shapes. In our analysis, we included only kymographs whose length was greater than 25 μm.

### 2.15. Measurement of OCR

Oxygen consumption rate (OCR) was measured in zebrafish larvae at 96-hpf with a Seahorse XF24 extracellular flux analyzer. *psen2^+/−^* fish were incrossed, and progeny larvae were placed into the XF24 microplate well (one larva per well) and blocked with a capture screen in the presence of 670 μL fish water (0.5 mM NaH2PO4, 0.5 mM NaHPO4, 3 mg/L instant ocean). Basal respiration was measured for 63 min at 28.5 °C, while the maximal respiration was measured upon administration of 0.5 μM carbonyl cyanide-4-phenylhydrazone (FCCP). A mixture of 2 μM rotenone (Rot) and 5 μM antimycin A was added to shut down the electron transport chain function, revealing the nonmitochondrial respiration. After the Seahorse analysis, larvae were genotyped.

### 2.16. Drug Treatment

The chemical compounds rapamycin (Merck KGaA, Darmstadt, Germany) and bafilomycin (Thermo Fisher Scientific, Waltham, MA, USA; Cat# PHZ1164) were used. Before drug administration, a hole was made in the chorion of 8-hpf embryos, whereas 24-hpf embryos were dechorionated. All drugs were dissolved in DMSO and stored in small aliquots at −20 °C. We performed 1 µM rapamycin treatment from 8- to 48-hpf and 40 nM bafilomycin treatment for 16 h prior to the end of the experiments, specifically either from 32- to 48-hpf or 16 h before 8-dpf. Starvation was induced starting from 6-dpf for two days. After treatments, embryos/larvae were collected in Eppendorfs, anesthetized, and vials were placed on ice. After 3 washes with cold PBS, embryos/larvae were deyolked in PBS with the addition of PMSF and centrifuged for 5 min at 4 °C at 5000 rpm. After centrifugation, supernatants were discarded, and samples were stored at −80 °C.

### 2.17. Protein Extraction and Western Blotting

Pools of 10 of 2- or 8-dpf *psen2^−/−^* and WT larvae, in basal conditions or upon treatments, were frozen in liquid nitrogen and mechanically homogenized in Ripa Buffer with the addition of protease (PIC, Cell Signalling, Danvers, MA, USA; Cat# 5871) and phosphatase inhibitors (PhosphoSTOP, Roche, Basel, Swiss; Cat# 4906837001). Following a brief centrifugation, the supernatant was collected, and the protein content was determined by Bradford assay. A 15 μg amount of total proteins per sample was boiled and loaded into 12% polyacrylamide precast gels (Thermo Fisher Scientific, Waltham, MA, USA; Cat# NP0341). Following SDS- PAGE, proteins were transferred onto a PVDF membrane (Thermo Fisher Scientific, Waltham, MA, USA; Cat# 88,520). This was subsequently saturated with 5% nonfat dry milk (Bio-Rad, Hercules, CA, USA; Cat# 1,706,404) in 1X Tris-buffered saline (Thermo Fisher Scientific, Waltham, MA, USA; Cat# 28,358) added with 0.1% Tween 20 detergent and hybridized with primary (anti-LC3 1:1000, Thermo Fisher Scientific, Waltham, MA, USA; Cat# PA1-16930; anti- btubulin 1:1000, Cell Signalling, Danvers, MA, USA; Cat# 2128) and secondary (HRP-conjugated goat anti-rabbit 1:2000). The signal was revealed with Immobilon Forte Western HRP substrate (Merck KGaA, Darmstadt, Germany).

### 2.18. Statistical Analysis

All data are representative of at least 3 independent experiments. All the results were expressed as means ± SEM. Data were analyzed by parametric and nonparametric tests to determine significant differences using the software GraphPad Prism version 8.00 for Mac OS X (La Jolla, CA, USA). Values of *p* < 0.05 were considered statistically significant. * = *p* < 0.05, ** = *p* < 0.01, *** = *p* < 0.001, **** = *p* < 0.0001.

## 3. Results

### 3.1. Generation of a psen2 Knockout (KO) Zebrafish Line

**Figure 1:**
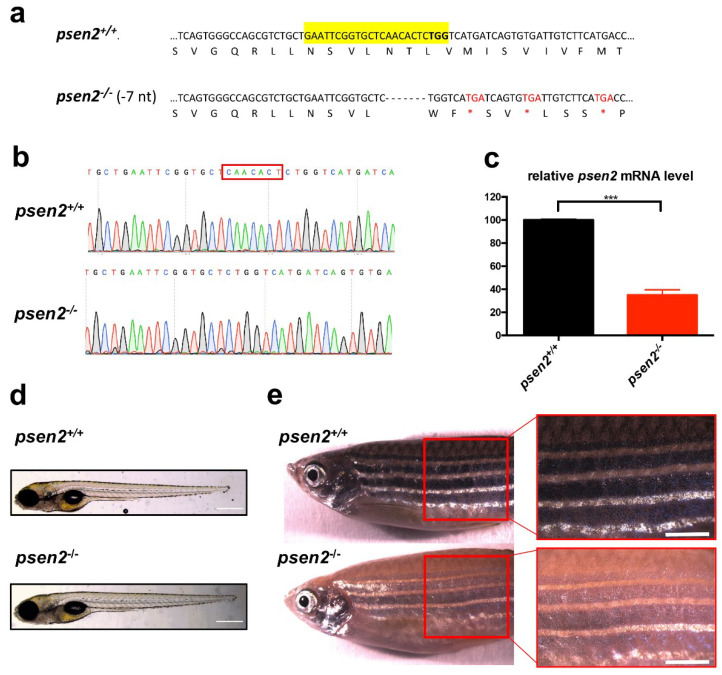
Knockout (KO) of *psen2* gene. (**a**) Partial nucleotide and amino acid sequences of *psen2* exon 4 showing a 7-bp deletion, confirmed by DNA sequencing. Deleted nucleotides are represented by dashes. CRISPR target site is highlighted in yellow and the protospacer-adjacent motif in bold. The 7-bp deletion results in a coding frameshift, with the insertion of premature stop codons (three in exon 4 marked in the figure as red asterisks, nine more in the following coding region). (**b**) Sequencing chromatographs of *psen2^+/+^* and *psen2^−/−^* alleles. The red box delineates the area of the induced deletion. (**c**) RT-qPCR analysis on 1-year *psen2^+/+^* and *psen2^−/−^* fish brain, showing a statistically significant decrease in *psen2* mRNA level in *psen2*^−/−^ mutants. Values represent the mean ± SEM. Data were generated from 3 biological replicates for each condition. Statistical significance was determined by two-tailed Student’s *t*-test (***, *p* < 0.001). (**d**) Representative images of *psen2^+/+^* and *psen2^−/−^* 6-dpf larvae. Scale bar: 500 µm. (**e**) *psen2^+/+^* and *psen2^−/−^* surface pigmentation at 6 months, showing an undetectable deposition of melanin in melanocytes of the *psen2^−/−^* mutant. Scale bar: 1 mm.

To generate a *psen2* KO zebrafish line, we mutated the zebrafish *psen2* gene using a CRISPR-Cas9 approach [27]. The CRISPR sequence was designed on its fourth exon (corresponding to the third coding exon). The sgRNA (Figure 1a) was injected together with the Cas9 mRNA. A heterozygous F1 carrier with a 7-nt deletion was selected from the offspring of F0 outcrosses and used to obtain the F2 generation. The deletion gave rise to a frameshift mutation that leads to the insertion of several premature stop codons starting from the amino acid 142 of the protein (Figure 1a,b). The putative encoded protein contains 141 amino acid residues of the whole 441 amino acid long zebrafish Psen2 protein. Hereafter, the obtained mutant line is referred to as *psen2*^ia52/ia52^ in the ZFIN database and hereafter.

Firstly, we investigated whether the introduced mutation resulted in quantitative changes in the *psen2*-transcribed products by RT-qPCR. *psen2* expression levels in the brains of 6-month post-fertilization (mpf) mutants, and relative controls, were analyzed (Figure 1c). A statistically significant reduction of *psen2* transcript levels to less than 35% of those measured in WT fish was observed, an outcome consistent with nonsense-mediated mRNA decay due to premature stop codon presence. Homozygous *psen2^−/−^* embryos, obtained by heterozygous mating, segregate according to a mendelian ratio, as revealed by the genotyping analysis of single larvae (both at 3- and 6-day post-fertilization (dpf)). Moreover, the offspring obtained from incrossed homozygous *psen2^−/−^* mutants, raised to adulthood, are viable and fertile. This excludes that the survival of homozygous fish, derived from heterozygous females, could be primarily due to the compensative effects of maternal *psen2* transcripts or proteins. During the first stages of development, the mutant line is phenotypically similar to WT siblings (Figures S1 and S2), but starts to lose surface melanotic pigmentation during larval-to-adult transition (Figure 1d,e). This defect, however, does not impact the retinal pigmented epithelium. While melanin deposition seems to be totally absent in the adult mutant fish, a faint striping is still visible, suggesting that melanocytes are present in the skin of *psen2^−/−^* adult animals, but they cannot produce melanin (Figure 1e). The pigmentation defect, that gradually develops in *psen2^−/−^* zebrafish, does not develop in heterozygous fish, a result in line with what has been observed in a previously generated *psen2* KO zebrafish line [25].

### 3.2. Psen2 Ablation Does Not Affect Notch Signaling at Early Developmental Stage

**Figure 2:**
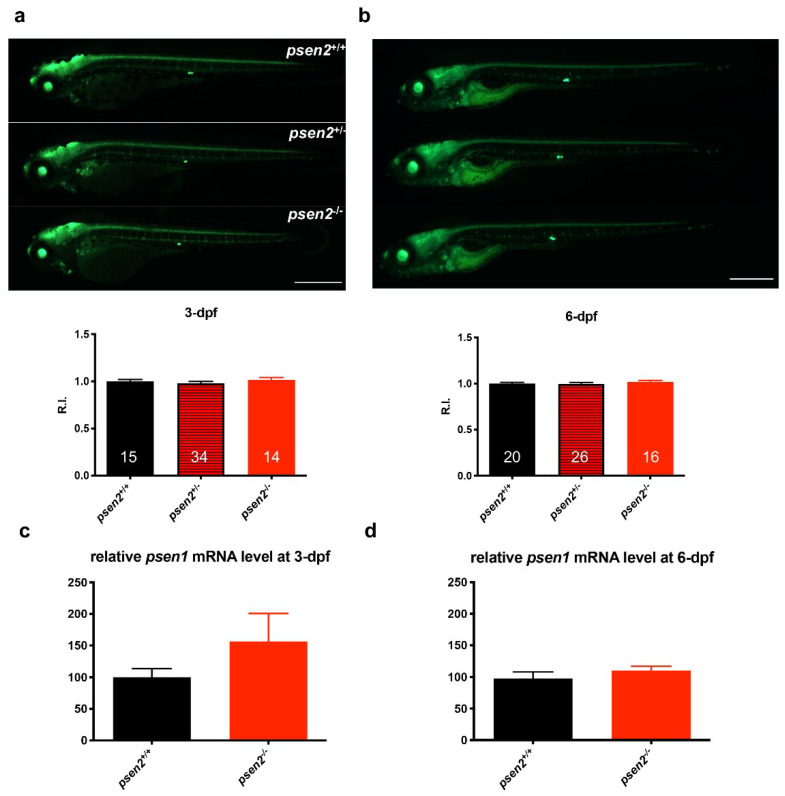
Effect of Psen2 absence on Notch signaling and *psen1* expression at early developmental stages. (**a**,**b**) Representative images and relative fluorescence quantification of *psen2^+/−^* incross progeny in *Tg(EPV.Tpi–Mmu.Hbb: EGFP)^ia^12* transgenic background, both at (**a**) 3- and (**b**) 6-dpf. Genotyping was performed after image acquisition. Values represent the mean ± SEM. Data were generated from 3 independent experiments. R.I. = Relative Intensity. Scale bar: 500 µm. Statistical significance was determined by one-way ANOVA test. (**c**,**d**) Quantification of relative *psen1* mRNA level in both (**c**) 3- and (**d**) 6-dpf *psen2^+/+^* and *psen2^−/−^* larvae. Values represent the mean ± SEM. Data were generated from 3 biological replicates for each condition. Statistical significance was determined by two-tailed Student’s *t*-test.

Given the role of Psen2-containing γ-secretase on Notch signaling [23], this latter was investigated in the *psen2^−/−^* line by crossing it with the reporter transgenic zebrafish line *Tg(EPV.Tpi–Mmu.Hbb:EGFP)ia12* [33]. The basic core Notch-transduction pathway is the same in most Notch-dependent processes: binding of the Delta ligand on one cell to the Notch receptor on another cell results in two proteolytic cleavages of the receptor. The second cleavage, carried out by γ-secretase, mediates the release of the Notch intracellular domain (Nicd), which enters the nucleus and acts as a transcription factor for responsive genes. The reporter line expresses a cytosolic eGFP under the control of Notch-responsive elements; thus, it produces the fluorescent protein depending on the processing and the activity of Notch and can be used to monitor the signaling pathway. Notch signaling was essentially unaltered in mutant larvae compared to controls, both at 3- and 6-dpf (Figure 2a,b), in line with the overall normal development of the mutant line (Figures S1 and S2). The result suggests an overall genetic compensation for this pathway, a phenomenon commonly observed when working with stable KO models, especially in zebrafish.

Genetic compensation may result from different mechanisms, including the upregulation of genes with redundant functions. In order to understand whether the mild phenotype developed by the mutant line was connected to a Psen1-mediated functional compensation of Psen2 ablation, we analyzed the expression level of psen1 in *psen2^−/−^* larvae, both at 3- and 6-dpf (Figure 2c,d). Overall, psen1 expression levels did not show significant alterations in *psen2^−/−^* larvae compared to controls, at both time points (although a slight increase is present at 3-dpf). This result suggests that the compensatory mechanisms sustaining γ-secretase-dependent Notch processing in the absence of Psen2 are not linked to a clear upregulation of psen1 transcription. Moreover, these results point to a dispensable role of Psen2 in Notch cleaving activities of γ-secretase during early larval stages.

### 3.3. Loss of Psen2 Alters Zebrafish Locomotor Behavior

To obtain more functional outcomes linked to the ablation of Psen2, we focused on behavioral assays. Indeed, while investigating neurological phenotypes, several studies have used motion tracking to characterize locomotor behavior in zebrafish larvae [34,35,36]. One of the most common behavioral assays is the vibrational startle response assay. Changes in startle reflex and habituation characterize abnormal sensory-processing functions and, hence, represent an informative index of neurophysiological health [37,38,39].

**Figure 3:**
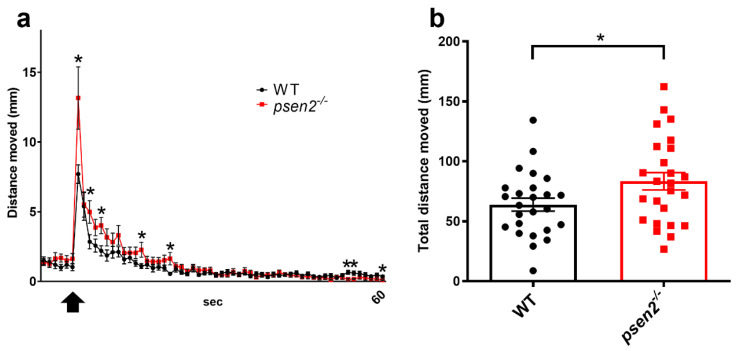
Effect of Psen2 absence on zebrafish larval behavior: vibrational startle analysis. (**a**) Plots of mean distance moved in response to a repetitive tapping stimulus in WT and *psen2^−/−^* larvae at 8-dpf. Black arrow indicates the beginning of tapping stimulation. (**b**) Quantification of the total distance moved by larvae during the entire experiment. Data were generated from 3 independent experiments. *n* = 25 larvae/condition. Values represent the mean ± SEM. Statistical significance was determined by two-tailed Student’s *t*-test (* = *p* < 0.05, ** = *p* < 0.01).

In our *psen2^−/−^* line at 8-dpf, the presentation of the first vibrational stimulus dramatically evoked an escape response, as it was for WT larvae, followed by an iterative reduction in startle response to stimulation, representing habituation. However, the response to the first stimulus in *psen2^−/−^* larvae was higher than in controls (Figure 3a). The increased motility in the initial steps of habituation and the overall increased distance moved by mutant larvae during the whole assay (Figure 3b) reflect the initial abnormal response to stimulation of the mutant larvae. Altogether, these data indicate an alteration in the sensory-processing functions of *psen2^−/−^* larvae, suggesting a functional role of Psen2 in the neurophysiology of zebrafish.

### 3.4. Psen2 Ablation Reduces Neuronal ER-Mitochondria Contacts But Not Mitochondrial Respiration

**Figure 4:**
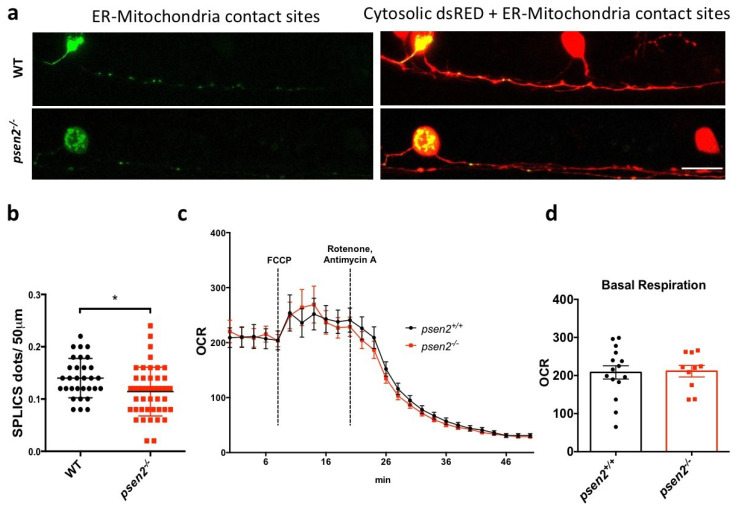
Effects of *psen2* genetic manipulation on in vivo ER-mitochondria juxtaposition and mitochondrial respiration. (**a**) Representative images of the transient expression of the SPLICS probe for ER-mitochondria contact sites in RB neurons of 1-dpf WT and mutant embryos. (**b**) Relative quantification of GFP reconstituted dots in the first 50 µm of RB axons in all the conditions analyzed. Data was generated from 3 or more independent experiments/conditions. Statistical significance was determined by two-tailed Student’s *t*-test. (**c**) Mean oxygen consumption rate (OCR) in *psen2^+/+^* and *psen2^−/−^* larvae at 4-dpf upon different treatments. Dashed bars indicate the addition of FCCP (0.5 µM) or Rotenone/Antimycin A (2 µM and 5 µM, respectively). Genotyping was performed after OCR measurements. WT: *n* = 15; *psen2^−/−^*: *n* = 10. Values represent mean ± SEM. (**d**) Quantification of basal OCR, as reported in (**c**). Statistical significance was determined by two-tailed Student’s *t*-test (* = *p* < 0.05).

Next, we focused our attention on another well-known cellular process modulated by PS2 in mammals, specifically the ER-mitochondria tethering [7,8,40]. To investigate organelle communication in vivo, the split GFP-based contact site sensor (SPLICS) was used. The SPLICS is a fluorescent probe exploiting the split GFP system to allow the visualization of different organelle contact sites [31,41,42], in our case ER-mitochondria ones. Specifically, by using the UAS/Gal4 binary system, we were able to transiently express the SPLICS probe in a specific subpopulation of zebrafish sensory neurons, called Rohon-Beard (RB) cells. WT and *psen2^−/−^* embryos in s1102t:Gal4 transgenic background were used, analyzing RB cells at 1-dpf. We decided to standardize ER-mitochondria contact site quantification by measuring SPLICS dots on the first 50 µm of RB axons. Indeed, the RB soma resulted in crowded mitochondria (Figure 4a), further complicating the analysis. At 1-dpf, *psen2^−/−^* RB neurons displayed a significantly reduced number of ER-mitochondria contact sites compared to WT controls (Figure 4a,b), as it was in cortical neurons from PS2–KO mice [40]. This result suggests a specific Psen2 involvement in the molecular machinery responsible for keeping the two organelles in contact, which cannot be compensated for in *psen2^−/−^* embryos.

ER-mitochondria communication highly impacts mitochondrial metabolism [43]; moreover, the recent data obtained in PS1/PS2-KO in vitro models reported a decreased mitochondrial respiration in the absence of these proteins. In this respect, we further characterized the *psen2* mutant line by performing an in vivo mitochondrial respiration analysis on whole 4-dpf larvae. In this case, however, both basal and maximal respiration were unchanged when compared to controls (Figure 4c,d), indicating an overall normal mitochondrial respiration in the absence of Psen2.

### 3.5. Psen2 Has a Key Role in Mitochondrial Axonal Transport

**Figure 5:**
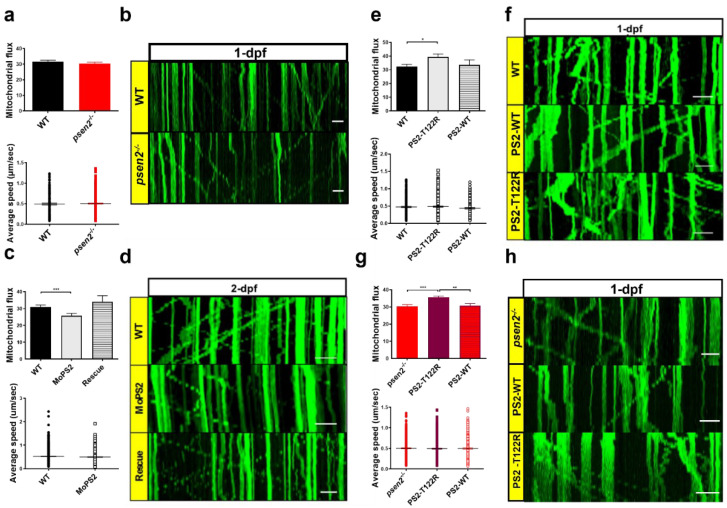
Effect of Psen2 genetic manipulation on mitochondrial axonal transport. (**a**) Mitochondrial flux (*n* of axons > 35/condition) and average speed (*n* of mitochondria > 200/condition) quantification in WT and *psen2^−/−^* 1-dpf zebrafish embryos. Data were generated from more than 5 independent experiments. (**b**) Representative kymographs showing mitochondrial movement in WT and *psen2^−/−^* 1-dpf embryos. Scale bar: 10 μm. (**c**) Mitochondrial flux (*n* of axons: WT, *psen2* morphants (MoPS2) > 50; rescue = 14) and average speed (*n* of mitochondria > 500 /condition) quantification in WT, *psen2* KD and *psen2* KD + human WT PS2 (Rescue) 2-dpf fish. Data were generated from more than 5 independent experiments. (**d**) Representative kymographs showing mitochondrial movement in WT, *psen2* morphants, and rescued fish at 2-dpf. Scale bar: 10 μm. (**e**) Mitochondrial flux (*n* of axons: WT = 44; + human PS2–T122R = 66; + human PS2–WT = 21) and mitochondrial average speed (*n* of mitochondria: WT = 320; + human PS2–T122R = 454; + human PS2–WT = 170) quantification in 1-dpf embryos, injected or not with human PS2 mRNA, WT or T122R. Statistical significance was determined by one–way ANOVA test (* = *p* < 0.05). Data were generated from more than 5 independent experiments. (**f**) Representative kymographs showing mitochondrial movement in 1-dpf WT fish, injected or not with mRNA of human PS2, WT or T122R, as indicated. Scale bar: 10 μm. (**g**) Mitochondrial flux (*n* of axons > 50/condition) and average speed (*n* of mitochondria > 300/condition) quantification in *psen2^−/−^* 1-dpf embryos injected or not with human PS2 mRNA, WT or T122R. Statistical significance was determined by one-way ANOVA test (* = *p* < 0.05, ** = *p* < 0.01, *** = *p* < 0.001). (**h**) Representative kymographs showing mitochondrial movement in 1-dpf *psen2^−/−^* fish, injected or not with human PS2 WT or T122R mRNA, as indicated. Scale bar: 10 μm.

ER-mitochondria tethering can influence several processes in the cell, such as autophagy, mitochondria dynamics, and organelle transport [44]. We, thus, took advantage of the use of zebrafish for investigating in vivo mitochondrial neuronal axonal transport in a WT or Psen2-null background (the *psen2^−/−^* line) and, upon human PS2, WT or AD mutant re-expression. Also in this case, we exploited the UAS–Gal4 system to retain the expression of a mitochondria-targeted Kaede protein (MT–Kaede) only in RB neurons of zebrafish embryos in the *s1102t:Gal4* transgenic background. Upon the expression of the fluorescent protein, RB axons of 1-dpf WT and mutant embryos were monitored at the confocal microscope, and the movements of their mitochondria were analyzed over time (Figure 5a,b). As reported, mitochondrial axonal transport (both the number of moving mitochondria over the number of total mitochondria in the axon, defined as mitochondrial flux, and their average speed) was not altered in 1-dpf *psen2^−/−^* embryos, compared to WT controls. This result suggests that the absence of Psen2 does not affect this cellular process at this developmental stage. However, since the occurrence of genetic compensation in a stable mutant line could influence the outcome of the experiment, we decided to perform the in vivo mitochondrial tracking in WT embryos upon the acute KD of *psen2*, obtained by the morpholino (MO) approach (Figure 5c,d). A significant decrease in mitochondrial flux in 2-dpf WT embryos after *psen2* KD was observed, without, however, affecting mitochondrial average speed. Importantly, this effect was reverted by rescue experiments (i.e., by re-expressing the human WT PS2 protein in *psen2*-KD, MO-treated, embryos). This demonstrates a Psen2-specific effect on this cellular process, which might be compensated for in *psen2^−/−^* embryos. Importantly, when the human AD–PS2–T122R mutant was expressed in WT embryos, a significant increase in the mitochondrial flux was observed (Figure 5e,f). This result suggests a gain-of-function of the PS2 mutant on this process. To deeply investigate this point, we used the *psen2^−/−^* pure background to test the effect of the expression of human PS2 (WT or T122R) on this process. Also in this case, the analysis reported a significant increase in mitochondrial flux upon the expression of the sole AD-PS2 mutant (Figure 5e). The expression of PS2–WT was, in fact, without effect (Figure 5f). The increase in mitochondrial flux caused by the expression of PS2–T122R involved both anterograde and retrograde mitochondrial transport (Figure S3a) and did not depend on an altered mitochondrial speed (Figure S3b) or density (Figure S4). Given the similar effect (increased mitochondrial flux) observed after the injection of PS2–T122R mRNA, in both WT and KO backgrounds, we can conclude that PS2 impacts axonal mitochondrial transport, although the expression of the protein was below the detection threshold of Western blot analysis.

Altogether, these results point to a direct involvement of PS2 in mitochondrial axonal transport, with a potentiation of the process in the presence of AD mutations. Since mitochondrial axonal transport could be influenced by organelle fission/fusion balance, mitochondrial morphology was also investigated in the different conditions analyzed. Organelle morphometric analysis (i.e., form factor and aspect ratio) did not show differences between WT and *psen2^−/−^* mitochondria (and between *psen2^−/−^* and *psen2^−/−^* + AD–PS2–T122R organelles) (Figure S5). This result suggests that the effects of AD–PS2 on mitochondrial axonal transport are not a consequence of altered mitochondrial morphology.

### 3.6. Psen2 Absence Causes a Dysregulated Autophagy

**Figure 6:**
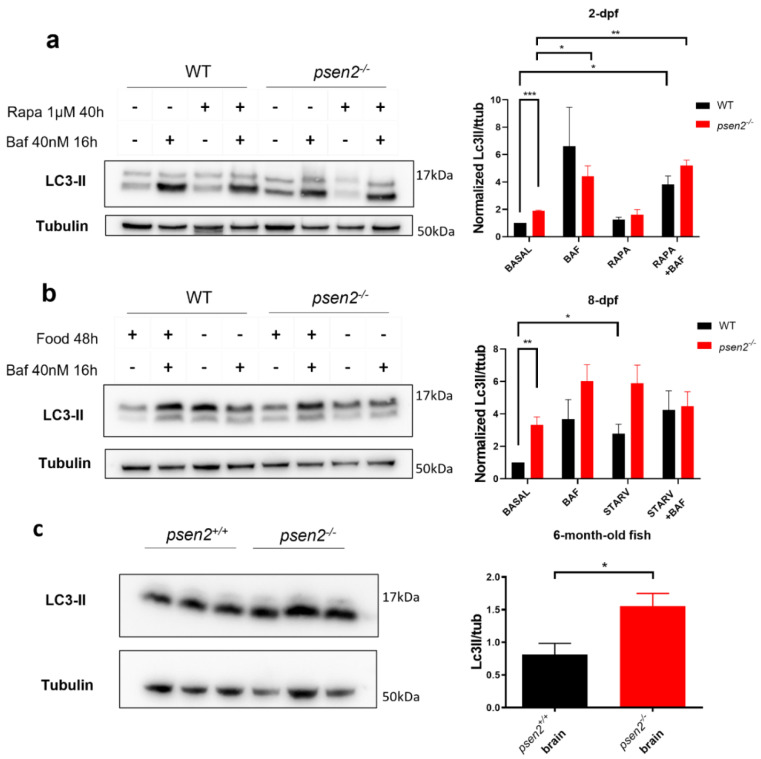
Effect of Psen2 absence on autophagy. (**a**,**b**) Representative blots and relative quantifications of LC3-II levels (normalized to tubulin levels) in WT and *psen2^−/−^* embryos or larvae at (**a**) 2- and (**b**) 8-dpf, respectively, in basal conditions or upon the induction of autophagy (by rapamycin, RAPA), in the presence or not of the lysosomal inhibitor bafilomycin (BAF). (**a**) Pools of 15 WT and *psen2^−/−^* embryos were treated with either vehicle (DMSO) or 1 µM Rapamycin (Rapa) from 8- to 48-hpf (to induce autophagy), with or without the addition of 40 nM Bafilomycin (Baf) at 32-hpf (final 16 h-treatment with the lysosomal inhibitor). Mean ± SEM, *n* = 3 independent experiments. (**b**) Pools of 10 6-dpf WT and *psen2^−/−^* larvae either fed (3 times per day) or starved for 2 days, treated or not with 40 nM BAF for the last 16 h of the experiment. Mean ± SEM, *n* = 3 independent experiments. (**c**) Representative blot and relative quantification of LC3-II levels (normalized to tubulin levels) in the brains of *psen2^+/+^* and *psen2^−/−^* 6-month-old fish. Data were generated from 3 brains for each genotype. Values represent the mean ± SEM. * = *p* < 0.05; ** = *p* < 0.01; *** = *p* < 0.001.

Autophagy is another cell process that could be linked to MAM modulation [45]. Considering the key role of PS2 in maintaining the correct communication between ER and mitochondria in mammalian cells [7,40,46], here reported also for Psen2 in zebrafish (Figure 4), and the involvement of the protein in multiple steps of the autophagy flux [16], we decided to investigate this catabolic process in the *psen2^−/−^* zebrafish line. The expression level of the autophagy marker LC3 was thus investigated in pools of larvae, both at 2- and at 8-dpf, by quantifying the level of the lipidated form LC3-II [47]. At 2-dpf (Figure 6a), the overall autophagic flux in *psen2^−/−^* embryos was similar to that observed in control fish and proceeded normally. The level of LC3-II, as reported, increased substantially upon the treatment with an autophagic flux inhibitor (bafilomycin). However, in mutant embryos, an increased LC3-II level in basal condition was found compared to WT fish (Figure 6b). This indicates a higher level of autophagy in the absence of Psen2. Similarly, at 8-dpf (Figure 6b), the autophagic flux did not appear blocked in mutant larvae (since it increased upon bafilomycin treatment or starvation) while, again, an increase in the LC3-II levels was observed in basal conditions in *psen2^−/−^* larvae compared to controls. Altogether, these results suggest an increase in autophagy in the absence of Psen2 during development and later stages of zebrafish life. This conclusion is further supported by the quantification of LC3-II levels in total brain homogenates of 6-month-old WT and *psen2^−/−^* fish, reporting a significantly higher protein level in the mutant brains (Figure 6c).

## 4. Discussion

The mechanisms underlying PS2 functions, and their potential effects in neuronal physiology and pathology, are still unclear. Here, we have generated a *psen2^−/−^* zebrafish line by CRISPR₋Cas9 technology to shed light on these aspects. The advantages of using *Danio rerio* as a model organism to study this protein include the fact that the fish possess an ortholog for both human PS2 and PS1. The homozygous *psen2^−/−^* mutant is viable and fertile, and shows, as the most severe phenotypic trait, a pigmentation defect involving melanocytes. Specifically, this defect develops during the larval-to-adult transition. This finding is in accordance with previous data from the literature [25] and could be explained by the fact that melanosome biogenesis seems to be Ca^2+^-dependent [48]. The roles of mammalian PS2 in modulating several intracellular Ca^2+^ pathways [49], in fact, are well known. Moreover, it has been shown that one of the PS2-containing γ-secretase substrates is the premelanosome (PMEL) protein, a molecule involved in melanosome maturation and melanin deposition [4].

Although the acute KD of *psen2* in zebrafish embryos led to a phenotype clearly linked to aberrant Notch signaling [24], the constitutive *psen2* KO did not markedly influence this signaling pathway nor cause overt developmental defects. We do not rule out, however, the possibility that subtle Notch-linked effects could be present in the mutant. Likely, compensatory mechanisms sustaining γ-secretase-dependent Notch signaling occur during the development in the *psen2^−/−^* line These mechanisms are, however, independent of an upregulation of the paralog gene *psen1*. Alternatively, Psen2 might be needed for γ-secretase-mediated cleavage of PMEL but dispensable for the release of the Notch intracellular domain.

Functionally, Psen2 depletion induces some behavioral defects in zebrafish. Specifically, mutant larvae showed a significantly exaggerated response following initial stimulation. This result, together with that of an unchanged muscle structure and development, hints at a possible neuronal hyperexcitability phenotype of *psen2^−/−^* larvae, that, however, needs further investigation to be confirmed. Notably, a feature that characterizes cortical neurons of PS2-KO mice is indeed hyperexcitability [40]. This might suggest a possible common involvement of the protein in both animals’ neurophysiology.

In mammalian cells, PS2 has multiple roles independent of its activity within the γ-secretase complex [50]. Several studies indicate its involvement in diverse cellular processes, such as intracellular Ca^2+^ homeostasis [49] and ER-mitochondria communication. On this latter aspect, PS2 (both WT and AD mutants) is able to modulate organelle apposition [7] by being enriched at MAMs and interacting with MFN2 [8], a master regulator of ER-mitochondria tethering [9]. The modulation of MAMs, in turn, can have different consequences. These domains of close apposition between ER and mitochondria, in fact, are required for the regulation of several cell processes, such as organelle Ca^2+^ transfer, lipid synthesis, mitochondrial dynamics and transport, and autophagy [46,51]. We thus took advantage of the newly generated zebrafish *psen2^−/−^* line to study the role of Psen2 in the ER-mitochondria tethering in vivo. We observed a significant decrease in organelle contacts in *psen2^−/−^* embryos at 1-dpf compared to controls. This result agrees with what has been observed in primary cortical neurons of PS2–KO mice [40] and in KD for PS2 in mammalian tissue culture [7].

Since ER-mitochondria connection is strongly linked to mitochondrial metabolism and this latter is altered in AD [52], specifically in different AD-PS2-related models [17], mitochondrial respiration was also investigated in the *psen2^−/−^* zebrafish line. Both basal and maximal respiration resulted comparable to those of WT fish. This result disagrees with what has been found in mouse PS2-KO primary neurons, where a minor defect in these parameters occurs [40]. A possible explanation for this discrepancy could be that Psen2 ablation may have a stronger tissue-specific respiratory effect on neurons, which was not detected when analyzing whole zebrafish larvae. Further experiments are required in order to clarify this aspect.

Given the connection between ER-mitochondria tethering and mitochondrial dynamics, we further characterized the *psen2^−/−^* zebrafish line by investigating mitochondrial axonal transfer in vivo. We analyzed WT zebrafish embryos, either KD for *psen2* (by the morpholino approach) or expressing the human PS2 protein, WT or carrying the AD-linked T122R mutation. We compared them with *psen2^−/−^* embryos themselves or re-expressing one or two forms of the human protein. In the *psen2* KD condition, mitochondrial axonal flux was decreased (and rescued by the re-expression of the human WT protein), while it was increased only when the AD–PS2–T122R mutant was expressed. These results suggest a gain-of-function of the mutant protein, compared to the WT form, in this process. Conversely, *psen2* KO embryos showed an unaltered mitochondrial flux with respect to controls, which could be explained by the occurrence of compensatory mechanisms in a condition of “stable ablation” of the protein. However, in the same mutant embryos, mitochondrial flux resulted significantly increased upon AD–PS2–T122R re-expression, in accordance with the data obtained overexpressing the same mutant in WT fish. The potentiated mitochondrial flux linked to AD–PS2 is not dependent on organelle distribution or biogenesis, as well as on their morphology and dynamics. Indeed, recently it was shown that primary cortical neurons from PS2-KO mice show an unchanged morphology of the neuronal mitochondrial network [40], reinforcing the results obtained in *psen2^−/−^* embryos.

Another cellular process modulated by PS2 in mammalian cells is autophagy, a pathway linked to ER-mitochondria communication and found altered in AD [53]. In particular, AD–PS2 mutants have been shown to block autophagy flux in different cell types [16]. Interestingly, PS2–containing γ-secretase complexes have been described as selectively targeted to late endosomes and lysosomes, while PS1-containing enzymes resulted more broadly distributed within the cell [4]. In this context, the specific intracellular distribution of PS2 compared to PS1 may be responsible for their partially different roles in the autophagy pathway, as reported in the literature [54]. To better understand the link between PS2 and autophagy, we investigated this catabolic process in the mutant *psen2^−/−^* line. The analysis did not reveal a blockage in the autophagic flux in *psen2^−/−^* larvae, both at 2- and 8-dpf. However, an increased basal autophagy in *psen2^−/−^* background compared to control is present. These results indicate a more pronounced autophagy in the absence of Psen2 since early developmental stages.

Interestingly, a potentiated autophagy, such as the one induced by rapamycin, has been linked to the inhibition of melanogenesis [55]. The link between melanogenesis and autophagy has already been established: autophagy is involved in both melanocyte biogenesis and degradation of melanosomes. Melanosomes are lysosome-related organelles in which melanin is synthesized [56]. However, discordant data have been collected on the topic, probably due to the great mechanistic complexity of melanogenesis regulation. Nonetheless, the fact that the *psen2^−/−^* line gradually accumulates melanocytic defects could be linked to an occurring dysregulation of autophagy. This idea is reinforced by the fact that a significant increase in LC3-II levels is detected in 6-mpf *psen2^−/−^* fish brain compared to WT controls. It is worth noting, however, that the pharmacological inhibition of γ-secretase for 15 days in adult zebrafish was found to decrease melanophore number by 40% [57]. Melanocyte survival was specifically linked to a proper γ-secretase-dependent Notch signaling [24]. However, this pathway resulted generally unaltered in our *psen2^−/−^* larvae, and the defective pigmentation affecting the mutant line seems to involve melanin deposition and not melanocyte maintenance itself. Thus, it is interesting to speculate on a possible γ-secretase-independent effect of Psen2 in melanosome homeostasis. Of note, however, pigmentation of PS2-KO mice did not show overt abnormalities [40]. On this aspect, development of the melanocyte lineage in mammals (reviewed [58]) is similar to that in zebrafish, with some notable exceptions. Indeed, once melanin is formed in mammalian melanocytes, it is packaged into melanosomes and transferred to other cells, such as keratinocytes of developing hair or neighboring epidermal cells. Instead, fish melanocytes retain their melanosomes, which can be redistributed throughout the cell [59]. Thus, the interspecies differences between mammals and *Danio rerio* [60] may underlie the differential effect of Psen2 ablation on pigmentation.

Additionally, an interesting paper by Daniele et al. reported that, in mouse melanocytes, mitochondria and melanosomes are physically connected, and this connection seems to be linked to melanogenesis. The physical contact between these two organelles, in fact, seems to be potentiated by acute stimulation of melanosome biogenesis, and conversely reduced in conditions of abnormal melanosome biogenesis [61]. The fact that melanosome-mitochondria communication seems to be mediated by MFN2, a well-known interactor of PS2 [8], further reinforces the involvement of the protein in melanocyte homeostasis. However, the mechanisms of Psen2 action in this process remain elusive. Finally, it should be highlighted that *psen1^−/−^* zebrafish does not develop a pigmentation defect [62], and that PS1 does not interact with MFN2 [8], thus implying a Psen2-specific effect on this pathway, further corroborating the nonredundant roles of Psen proteins in zebrafish physiology.

## Data Availability

Data are contained within the article or supplementary material.

## Supplementary Materials

The following supporting information can be downloaded at: https://www.mdpi.com/article/10.3390/cells12030376/s1, Figure S1: Effect of Psen2 absence on neuronal and heart development; Figure S2: Effect of Psen2 absence on muscle development; Figure S3: Effect of human AD–linked PS2–T122R expression on mitochondrial axonal transport in *psen2^−/−^* embryos; Figure S4: Effect of Psen2 genetic manipulation on mitochondrial density; Figure S5: Effect of Psen2 genetic manipulation on mitochondrial morphology.
