# Supplementary material for: Unraveling Presenilin 2 Functions in a Knockout Zebrafish Line to Shed Light into Alzheimer’s Disease Pathogenesis
Source: Cells. 2023 Jan 19;12(3):376. doi: 10.3390/cells12030376 (PMC9913325; doi:10.3390/cells12030376)
Supplement: Supplementary file 1 [file cells-12-00376-s001.zip › cells-2155829-supplementary.pdf]

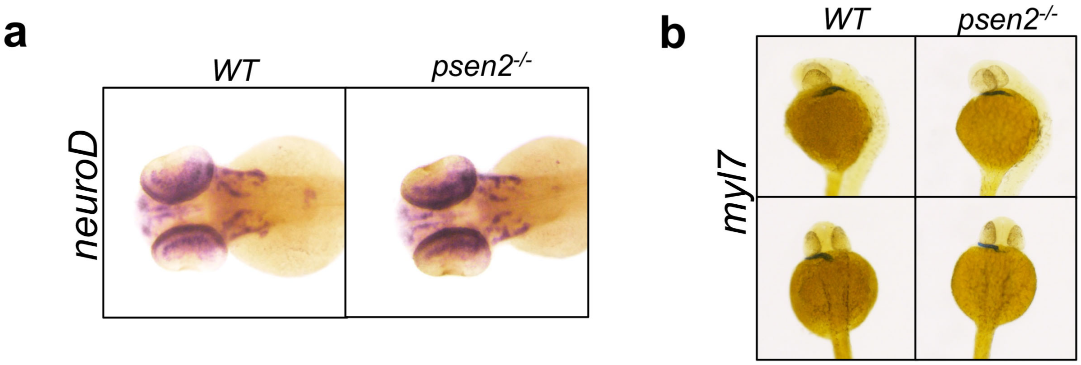

Figure S1. Effect of Psen2 absence on neuronal and heart development. Representative images of whole mount *in situ* hybridizations against *neuroD* **(a)** and *myl7* **(b)**, at 48-hpf and 24-hpf, respectively, performed in both WT and *psen2*$^{-/-}$ embryos. More than 15 embryos per conditions were analysed. Scale bar: 250 µm.

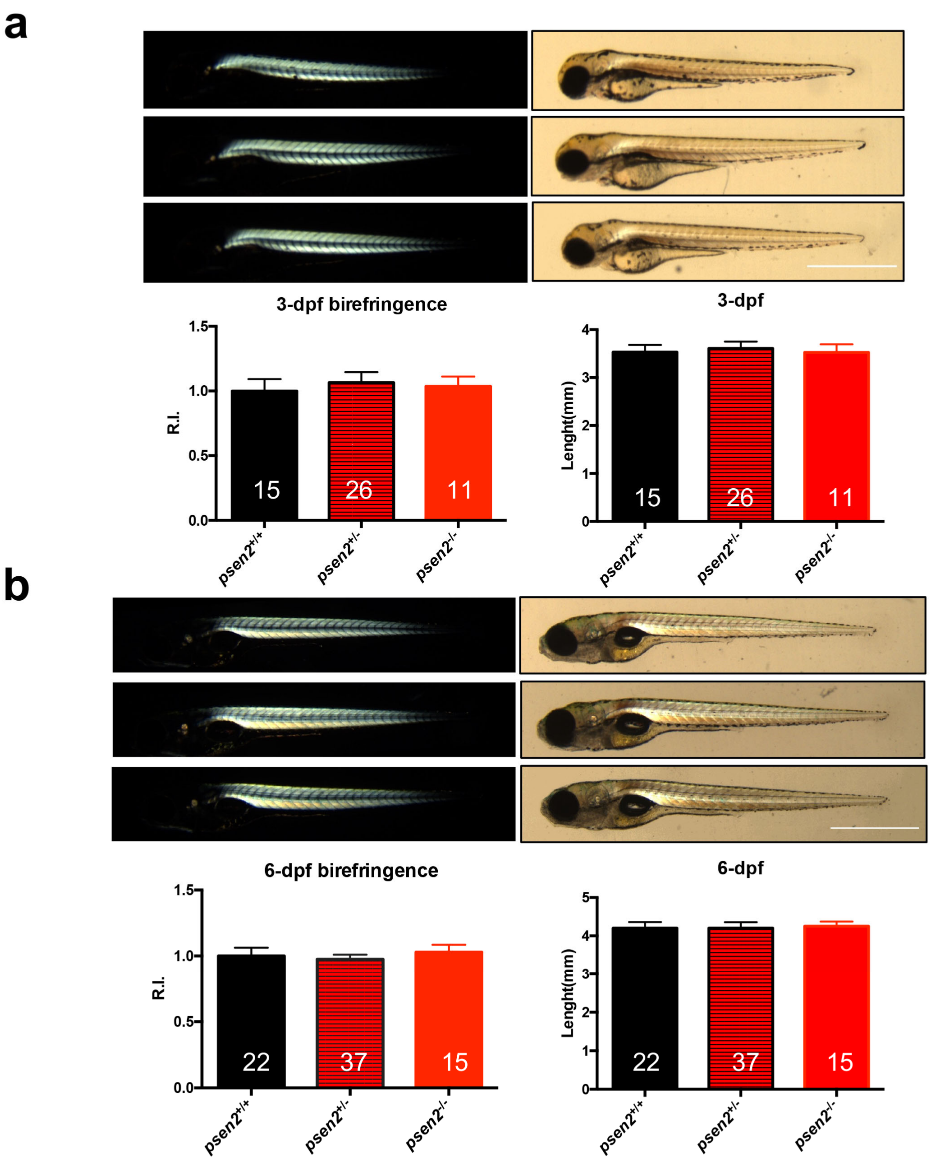

Figure S2. Effect of Psen2 absence on muscle development. Representative images and relative quantifications of birefringence analyses and fish length performed both at 3- **(a)** and 6- **(b)** dpf in *psen2*$^{+/-}$ incross progeny. Genotyping was performed after birefringence analysis. Values represent the means ± SEM. Data were generated from 3 independent experiments. Scale bar: 1 mm. Statistical significance was determined by One-way Anova test.

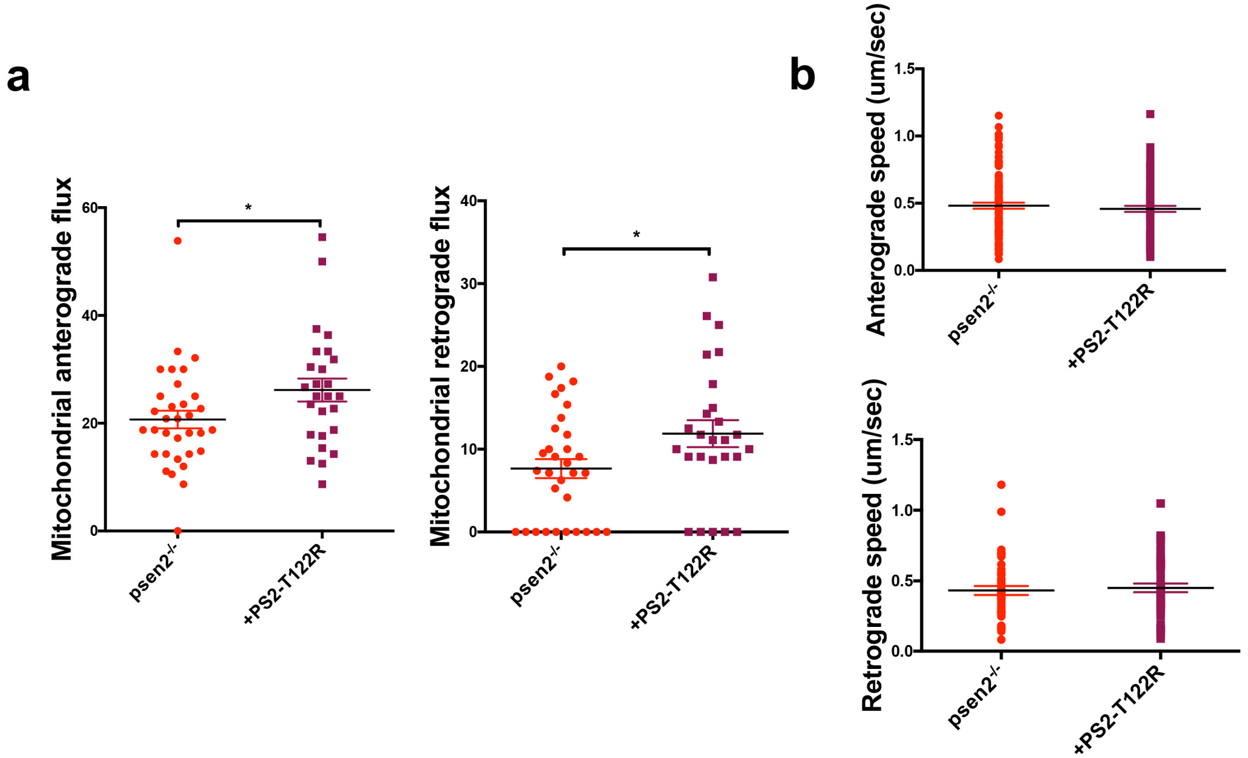

Figure S3. Effect of human AD-linked PS2-T122R expression on mitochondrial axonal transport in *psen2*$^{-/-}$ embryos. Anterograde and retrograde mitochondrial flux (n of axons > 25/condition) and directional average speed (n of mitochondria > 50/condition) in *psen2*$^{-/-}$ embryos, injected or not with the human PS2-T122R mRNA. Statistical significance was determined by two tail Student's t test (*= P<0.05). Data were generated from more than 3 independent experiments.

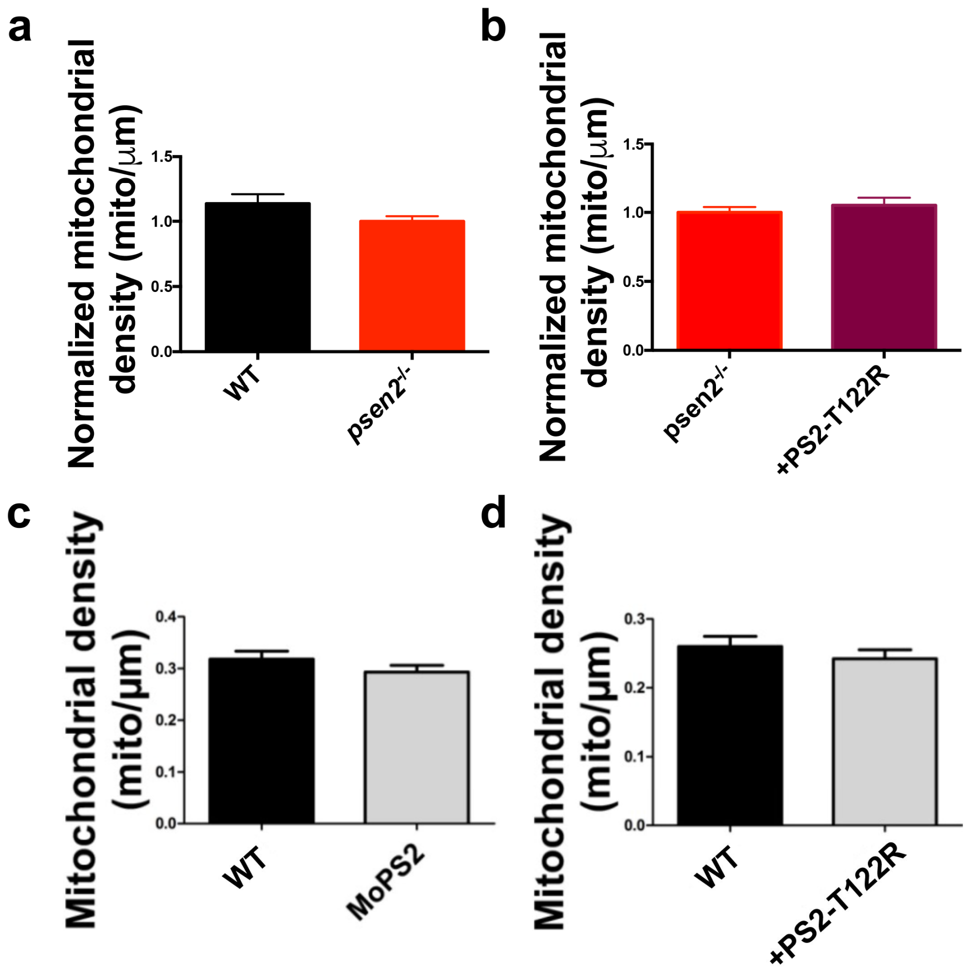

Figure S4. Effect of Psen2 genetic manipulation on mitochondrial density. a, b) Mitochondrial density quantified in 1-dpf embryos, WT compared to *psen2*$^{-/-}$ ones **(a)**, and *psen2*$^{-/-}$ compared to *psen2*$^{-/-}$expressing PS2-T122R ones (b). Statistical significance was determined by two tail Student's T test. Values represent the mean ± SEM. WT: N= 60 axons; *psen2*$^{-/-}$: N= 46 axons; *psen2*$^{-/-}$ + PS2-T122R: N= 32 axons. **c, d)** Mitochondrial density in WT and upon *psen2* KD **(c)** or human AD-linked PS2-T122R expression **(d)** in 2- or 1-dpf embryos, respectively. **c)** N= 20 for each condition, d) WT: N= 51, WT + PS2-T122R: N= 36.

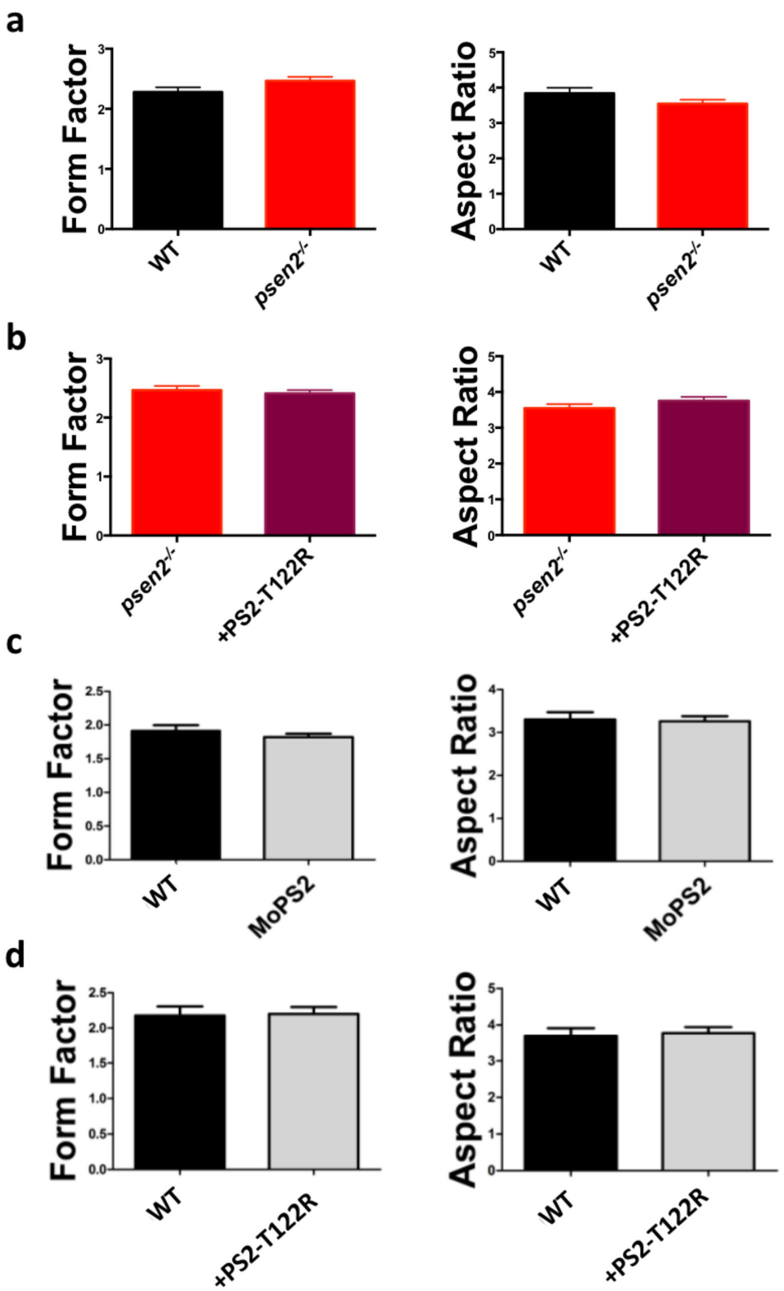

Figure S5. Effect of Psen2 genetic manipulation on mitochondrial morphology. a, b) Mitochondrial morphology, as reported by the quantification of Form factor and Aspect ratio, in 1-dpf WT and *psen2*$^{-/-}$ embryos **(a)** or upon human AD-linked PS2-T122R expression **(b).** Statistical significance was determined by two tail Student's T test. Values represent the mean ± SEM. WT: N= 26 axons; *psen2*$^{-/-}$: N= 34 axons; *psen2*$^{-/-}$+ PS2-T122R: N= 31 axons. **c, d)** Mitochondrial morphology in WT embyos upon *psen2* KD at 2-dpf **(c)** or upon human AD-linked PS2-T122R expression at 1-dpf **(d)**. N= 20 axons per condition.
